# A modified EDAS model for comparison of mobile wallet service providers in India

**DOI:** 10.1186/s40854-022-00443-5

**Published:** 2023-01-19

**Authors:** Sanjib Biswas, Dragan Pamucar

**Affiliations:** 1 Decision Sciences and Operations Management, Calcutta Business School, Bishnupur, South 24 Parganas, West Bengal 743503 India; 2 Department of Operations Research and Statistics, Faculty of Organizational Sciences, University of Belgrade, 11000 Belgrade, Serbia

**Keywords:** Picture fuzzy, Grey correlation, EDAS method, TOPSIS, Comparison of mobile wallets

## Abstract

The present paper has two-fold purposes. First, the current work provides an integrated theoretical framework to compare popular mobile wallet service providers based on users' views in the Indian context. To this end, we propose a new grey correlation-based Picture Fuzzy-Evaluation based on Distance from Average Solution (GCPF-EDAS) framework for the comparative analysis. We integrate the fundamental framework of the Technology Acceptance Model and Unified theory of acceptance and use of technology vis-à-vis service quality dimensions for criteria selection. For comparative ranking, we conduct our analysis under uncertain environments using picture fuzzy numbers. We find that user-friendliness, a wide variety of use, and familiarity and awareness about the products help reduce the uncertainty factors and obtain positive impressions from the users. It is seen that PhonePe (A3), Google Pay (A2), Amazon Pay (A4) and PayTM (A1) hold top positions. For validation of the result, we first compare the ranking provided by our proposed model with that derived by using picture fuzzy score based extensions of EDAS and another widely used algorithm such as The Technique for Order of Preference by Similarity to Ideal Solution. We observe a significant consistency. We then carry out rank reversal test for GCPF-EDAS model. We notice that our proposed GCPF-EDAS model does not suffers from rank reversal phenomenon. To examine the stability in the result for further validation, we carry out the sensitivity analysis by varying the differentiating coefficient and exchanging the criteria weights. We find that our proposed method provides stable result for the present case study and performs better as ranking order does not get changed significantly with the changes in the given conditions.

## Introduction

The present paper endeavours to put forth a comparative analysis of some of the popular M-Wallet service providers based on users’ opinions. The theoretical foundations of two widely used frameworks such as TAM and UTAUT and the findings of the past work are considered to select the attributes for comparing the M-Wallets based on subjective opinions of a group of users having different demographic backgrounds. For comparative analysis, the existing algorithm of EDAS method is modified using grey theory and picture fuzzy logic. In the aftermath of the revolution in the field of wireless communication technology supported by significant development in hardware and computing domain, worldwide, mobile phone usage has increased massively over the last decades. Increasing mobility in communication, convenience, and advanced services has made mobile phones an integral part of human lives, significantly influencing all spheres of life (Jack and Suri 2011; Thakur and Srivastava 2014; Aydin and Burnaz 2016; Madan and Yadav 2016). The rapidly increasing rate of multi-faceted usage of mobile phones (i.e., smartphones) has presented vast opportunities for technology-based firms in various sectors and has accelerated the growth in mobile technology-based business solutions (Flood et al. 2013; Petter et al. 2013; Kuganathan and Wikramanayake 2014; Attour et al. 2015; Viswanathan et al. 2017). Among various such technology-based services, mobile phone payments have increased significantly in recent years. M-Wallet services have drawn interest from a sizeable number of consumers. The number of service takers is increasing daily, accepting and adopting M-Wallets as an alternative way to pay electronically at a place they prefer and at the time they want, without reaching the point of sale physically (Duncombe and Boateng 2009; Leavitt 2012; Dennehy and Sammon 2015; Tang et al. 2014).

The usage is not restricted to age and profession, though a general notion is that tech-savvy, educated, and young consumers usually prefer to use M-Wallets. The recent outbreak of COVID-19 has acted as a catalyst to unleash the importance of M-Wallets to run daily activities for households and businesses. Extreme disruption and a stringent requirement of maintaining health and hygiene (minimization of payments using cash to prevent spreading infections) have resulted in a surge in the usage of M-Wallets. The rise in the number of M-Wallet users during the last year stands as a distinct outlier worldwide compared with yearly historical data prior to 2020 (Nandi and Banerjee 2020). A recent study (Statista Mobile POS Payments xxxx) reported that the expected number of mobile POS payers would be 1754.6 million by 2024.

During the last several years, the GOI has emphasized achieving its financial inclusion goal. The GOI aims to leverage the electronic medium for payment for the inclusion of a large part of the society (consists of small traders and merchants, people working in un-organized sectors, and low-literate people) into the mainstream, given the favourable rate of penetration of mobile phones in both urban and rural areas.^1^^,^ 2 However, the upward trend in the use of mobile payments started in India post-2014 with two significant initiatives taken by the GOI, such as “Digital India” and “Demonetization.” The decision to phase-wise shift towards a new regime for moving forward with paper-free, cashless and virtual operations with various initiatives, supported by incentives to Fin-Tech companies; development of faster wireless networks and mass-promotion of new innovative technologies have created a conducive environment for the growth of M-Wallet service providers (Kumar et al. 2011; Chattopadhyay et al. 2017; Sinha et al. 2019; Mittal and Kumar 2018). After the declaration of demonetization on November 08, 2016, India has witnessed a rapid enhancement in the consumer base using online payments in successive years (Padiya and Bantwa 2018). In effect, it has been observed that mobile phones are being used in various financial services. As a result, the M-Wallet market flourished, and many new entrants (both from public and private initiatives) have come into the picture (Pal et al. 2019; Sharma and Kulshreshtha 2019; Liébana-Cabanillas et al. 2020a). In India, a recent publication (Asher 2020) estimated approximately 760 million smartphone users by 2021 and around 973 million in 2025 (in 2013, it was 76 million) against a global prediction of approximately 3.8 billion by 2021. There is a massive potential for the M-Wallet market.

Several researchers and practitioners have worked on this area, given the promising growth and future potential of the M-Wallet sector. The extant literature shows that contributors put effort into investigating why consumers use M-Wallet services and what factors influence their decision to select a service provider. In this regard, most of the past contributions used TRA (Fishbein and Ajzen 1975), TPB (Ajzen 1991), and TAM (Davis 1989). While TRA sheds light on behavioural intentions controlled by attitude and subjective norms (Fishbein and Ajzen 1975; Ajzen and Fishbein 1980), TPB extends the horizon by including “the perceived ease or difficulty of performing the behaviour,” assuming that every decision-maker is rational (Ajzen 1991). Using the foundations laid by TRA and TPB, TAM stands on two essential pillars, perceived ease of use and usefulness (Davis 1989), which gained more popularity among researchers than its predecessors, particularly for explaining consumer behaviour related to technology products (Hong et al. 2006). We notice the extensive application of TAM and its extension, UTAUT (Venkatesh et al. 2003) in understanding the motives and nature of the behaviour of the consumers for using M-Wallets and electronic payments. The studies in various countries, including India (Aydin and Burnaz 2016; Pousttchi and Wiedemann 2007; Chen and Nath 2008; Shin 2009; Yang et al. 2012; Liébana-Cabanillas et al. 2014, 2020b; Dwivedi et al. 2014; Kapoor et al. 2014, 2020; Phonthanukitithaworn et al. 2015; Yadav 2016; Manikandan and Jayakodi 2017; Mei and Aun 2019, Pal et al. 2020; Singh and Sinha 2020; Singh et al. 2020), revealed various factors influencing the decisions to use M-Wallets. Mobile technology solutions such as consumer attitude, expectancy about performance, innovative applications, facilitation, convenience, user-friendliness, speed of transaction, compatibility issues, social pressure, information security and privacy, trust, cost of operations, and rewards influence the consumers. Summarizing these studies, it is evident that researchers mostly attempted to work on influencing factors in a fragmented way and carried out causal analysis using regression, structural equation modelling, and other statistical tests using empirical methods.

### Motivation of the research

Within our best possible search, we have noticed that a holistic comparison of M-Wallet service providers based on multiple factors or criteria is rare. In one recent study (see footnote 2), the authors tried to do a comparative analysis of service providers using a semantic differential scale and entropy-based approach based on the opinions of the respondents belonging to a young target group (21–30 years) in an empirical setup. However, this work also does not reflect a comprehensive view as it emphasizes the service quality dimensions and attitude of the users. Further, response-based comparison suffers from impreciseness which cannot be captured effectively in a crisp domain. In addition, people belong to an age group of 31–40, and even more avail M-Wallet services. Therefore, it is quite imperative to consider their views also. In another recent study (Kapoor et al. 2020), the authors argued for developing appropriate service quality dimensions for comparing solution providers. They applied a fuzzy-TOPSIS framework. However, the authors did not compare service providers. In this paper, we use service quality parameters aligned with the basic framework of TAM and UTAUT used in past research to respond to the gaps noticed in the past work. We use these factors as criteria to compare some of the popular M-Wallet service providers. As Dahlberg et al. (Dahlberg et al. 2015) advocated for an appropriate ecosystem for holistic comparison, we follow a user opinion-based MCDA while considering dealing with impreciseness in information. Further, in this paper, we apply a widely used recent MCDA algorithm such as EDAS with imprecise information for user opinion-based comparison of M-Wallet services.

Compared with the VIKOR and TOPSIS, EDAS method also evaluates the alternatives based on their separation from the ideal or preferential point. However, instead of the distance from two extreme ideal points (i.e., positive and negative), in EDAS, the distances of the alternatives from the average solution (such as PDA and NDA) are calculated. The preferred alternative is identified based on higher PDA and lower NDA values. Since the EDAS method considers the average solution point as the yardstick, it is free from extreme point variation and decision-making fluctuations. Therefore, the EDAS algorithm operates well in an uncertain environment and can deal with various complex decision-making problems by providing better accuracy and aggregation (Ghorabaee et al. 2015, 2016). However, we have observed a scantiness of research work that combine two perspectives of uncertainty measures such as grey theory and picture fuzzy logic with classical EDAS method to provide a comprehensive MCDM based analysis.

### Contributions of the paper

Our paper contributes to the growing literature in two ways. First, it uses a combined theoretical framework for a holistic multi-criteria based comparison of M-Wallet service providers in India. The criteria are selected in tune with extant literature using the basic framework TAM and UTAUT with service quality considerations. Second, we provide a novel extension of the EDAS method in an uncertain environment using GC and PFNs. Thirdly, the present paper provides a rare combination of grey theory and picture fuzzy logic in conjunction with EDAS method that provides a greater flexibility and comprehensive uncertainty based model for the analysts in solving various real-life complex problems.

### Paper organization

The rest of the paper is presented as follows. The following section ("Related work" section) gives a summary of some of the related work. "Preliminaries: PFS and PFN" section provides some preliminary concepts on PFS and PFN. In "EDAS method" section, we discuss about the original EDAS algorithm. We explain the procedural steps of our proposed methodology in "Proposed methodology: grey correlational picture fuzzy EDAS (GCPF-EDAS)" section. "Case study: M-Wallet selection" section exhibits the data analysis related to our problem of this paper and obtained results. In "Validation and sensitivity analysis" section, discussions on the findings are included and finally, "Conclusion and future scope" section concludes the paper and highlights some of the future scope.

## Related work

The EDAS method have been used by several researchers to evaluate alternatives for solving various problems in both engineering and management domains. For example, researchers have applied EDAS method to solve the issues like supplier selection on environmental dimensions and order allocation (Ghorabaee et al. 2017a), comparison of bank performances (Ghorabaee et al. 2017b), project management (Feng et al. 2018), comparison of the third-party logistics service providers (Ecer 2018), solid waste disposal (Kahraman et al. 2017; Behzad et al. 2020), construction management (Stanujkic et al. 2017; Hasheminasab et al. 2019), investment decision-making (Karmakar et al. 2018), personnel selection problem (Stanujkic et al. 2018), manufacturing performance (Stevic et al. 2018), comparison of performance of steam boilers (Kundakcı 2019), material selection (Chatterjee et al. 2018), renewable energy management (Asante et al. 2020), and contractor evaluation (Ghorabaee et al. 2018a). One of the major reasons behind the popularity of the EDAS method is its freedom from the rank reversal problem, which occurs for the TOPSIS algorithm (Ghorabaee et al. 2018b). After its proposal, several researchers have contributed to extending the basic framework for the EDAS method over the last five years. The extant literature reveals the applications of a modified and extended framework of the EDAS method using fuzzy sets (Ecer 2018; Hasheminasab et al. 2019; Stevic et al. 2018), intuitionistic fuzzy sets (Kahraman et al. 2017), interval fuzzy sets (Ilieva 2018), dynamic fuzzy sets (Ghorabaee et al. 2018a), Interval type 2 fuzzy sets (Ghorabaee et al. 2017a, 2017c), hesitant fuzzy linguistic scale (Feng et al. 2018), normally distributed data in a stochastic environment (Ghorabaee et al. 2017b), neutrosophic fuzzy linguistic scales (Li et al. 2019), interval-valued neutrosophic sets (Karasan and Kahraman 2018a; Karaşan and Kahraman 2018), neutrosophic fuzzy soft set with new similarity measures (Peng and Liu 2017), q-rung orthopair fuzzy sets (Li et al. 2020), and interval grey numbers (Stanujkic et al. 2017).

In this context, Zhang et al. (Zhang et al. 2019) proposed a multi-criteria group decision making model based on EDAS using the PFN. The concept of PFS with basic operations and properties was introduced by Cuong and Kreinovich (Cuong and Kreinovich 2014, 2013). A typical PFS is expressed in terms of three kinds of membership functions such as positive, neutral and negative. In addition, it captures the degree of refusal also. PFS has been used in multi-criteria based analysis for solving various problems (for example, (Yang and He 2019)) and gradually got developed and extended over the years. For instance, Wei used picture fuzzy (PF) cross entropy (Wei 2016), defined various similarity measures (Wei 2017b, 2018a), and proposed different aggregation operators (Wei 2017, 2018b). In this context, Wei and Gao introduced a generalized dice similarity measure for PFS (Wei and Gao 2018). PFS has been used to extend the TODIM method (Wei 2018c), TOPSIS algorithm (Yang and He 2019) and propose a projection model (Wei et al. 2018). Some extensions such as picture fuzzy matrix (Dogra and Pal 2020a), m-polar PF algebra (Dogra and Pal 2020b) and PF sub-rings (Dogra and Pal 2021) are also evident in the extant literature. However, through the possible search we find that PFS has not been yet used widely to extend the existing MCDM algorithms. Further, for EDAS method, use of PFS is not done to a significant extent.

However, EDAS method suffers from a limitation that it uses distance as a measurement scale. The average point may not be decided precisely in a typical scenario which involves substantial amount of subjectivity (Das and Chakraborty 2022). Further, classical EDAS method many a times does not reveal true ranking when comparing the alternatives that are too similar in magnitude or differing from each other largely (Ilieva et al. 2018). Further, in a circumstance which involves degree of refusal (in case of PFS) along with degrees of positive, negative and neutral membership, there is some amount of information loss. The concept of grey theory (Julong 1982, 1989) is suitable to use when a significant amount of subjectivity and information loss is present and it becomes arduous to define the fuzziness (Chithambaranathan et al. 2015). In the domain of MCDA techniques and their applications in solving various problems, grey theory and related concepts have been used plenteously. The GC method is derived from the concept GRA which works on the fundamental principles of grey theory (Xia et al. 2015). GC finds its importance among the researchers as it can measure the strength of relations among the data sequences under varying condition and provides estimation even with low volume cases. As a result, grey concepts are used with fuzzy sets and multivariate models in tandem (Das et al. 2019; Chakraborty et al. 2018, 2019). The expanding literature shows numerous occasions wherein grey concepts are used in decision-making problems (Huang and Jane 2009), for instance, supplier selection (Badi and Pamucar 2020), product selection (Pamucar 2020), and materials selection (Chatterjee and Chakraborty 2012). Das and Chakraborty (Das and Chakraborty 2022) infused the concept of GC in the basic framework of EDAS for solving a production engineering problem. Here, we endeavour to integrate the models of Das and Chakraborty (Das and Chakraborty 2022) and Zhang et al. (Zhang et al. 2019) PF environment for presenting an opinion-based service provider selection framework.

## Preliminaries: PFS and PFN

In this section, we present some preliminary concepts pertaining to the domain of PFS and PFN.

### Definition

Let ${\tilde{\text{A}}}$ denotes a PFS on a universe of discourse U. Then, ${\tilde{\text{A}}}$ is defined as (Cuong and Kreinovich 2014, 2013)

1 $${\tilde{\text{A}}} = {\text{x}}, \upmu _{{{\tilde{\text{A}}} }} \left( {\text{x}} \right),\upeta _{{{\tilde{\text{A}}}}} \left( {\text{x}} \right),\upupsilon _{{{\tilde{\text{A}}}}} \left( {\text{x}} \right)$$

where ${\text{x}} \in {\text{U}};\upmu _{{{\tilde{\text{A}}}}} \left( {\text{x}} \right),\upeta _{{{\tilde{\text{A}}} }} \left( {\text{x}} \right),\upupsilon _{{{\tilde{\text{A}}} }} \left( {\text{x}} \right) \in \left[ {0,1} \right]$ are the degrees of positive, neutral and negative membership of x in ${\tilde{\text{A}}}$ respectively such that

2 $$0 \le\upmu _{{{\tilde{\text{A}}} }} \left( {\text{x}} \right) +\upeta _{{{\tilde{\text{A}}} }} \left( {\text{x}} \right) +\upupsilon _{{{\tilde{\text{A}}} }} \left( x \right) \le 1\quad \forall {\text{x}} \in {\text{U}}$$

PFS has been derived from the traditional fuzzy sets. Here, if $\upeta _{{{\tilde{\text{A}}}}} \left( {\text{x}} \right) = 0$ then it resembles the IFS and if both $\upeta _{{{\tilde{\text{A}}}}} \left( {\text{x}} \right) =\upupsilon _{{{\tilde{\text{A}}} }} \left( {\text{x}} \right) = 0,{\tilde{\text{A}}}$ becomes a classical fuzzy set. The neutrality component provides a clearer ‘picture’ of the information and enables to carry out a more granular analysis for improving accuracy (Wang et al. 2017). PFS has another component such as degree of refusal ($\uppi _{{{\tilde{\text{A}}} }} \left( {\text{x}} \right)$) which provides the decision-makers not to give opinions when they are not interested. Therefore, PFS is more efficient in capturing uncertainties.

3 $$\uppi _{{{\tilde{\text{A}}} }} \left( {\text{x}} \right) = 1 - \left( {\upmu _{{{\tilde{\text{A}}} }} \left( {\text{x}} \right) +\upeta _{{{\tilde{\text{A}}} }} \left( {\text{x}} \right) +\upupsilon _{{{\tilde{\text{A}}} }} \left( {\text{x}} \right)} \right)\quad \forall {\text{x}} \in {\text{U}}$$

For a given element x in U, a PFN is represented as

4 $${\text{A}} = \left\{ {\left\{ {\left( {\upmu _{{{\rm A}}} ,\upeta _{{{\rm A}}} ,\upupsilon _{{{\rm A}}} } \right)|\upmu _{{{\rm A}}} ,\upeta _{{{\rm A}}} ,\upupsilon _{{{\rm A}}} \in \left[ {0,1} \right]\quad {\text{and}}\quad 0 \le\upmu _{{{\rm A}}} +\upeta _{{{\rm A}}} +\upupsilon _{{{\rm A}}} \le 1 } \right\}} \right\}$$

The properties and operations are given below (Cuong and Kreinovich 2014, 2013).

### Properties

Let, ${\tilde{\text{A}}} = {\text{x}}, \upmu _{{{\tilde{\text{A}}}}} \left( {\text{x}} \right),\upeta _{{{\tilde{\text{A}}} }} \left( {\text{x}} \right),\upupsilon _{{{\tilde{\text{A}}} }} \left( {\text{x}} \right)$ and ${\tilde{\text{B}}} = {\text{x}}, \upmu _{{{\tilde{\text{B}}} }} \left( {\text{x}} \right),\upeta _{{{\tilde{\text{B}}} }} \left( {\text{x}} \right),\upupsilon _{{{\tilde{\text{B}}} }} \left( {\text{x}} \right)$ are two PFS $\forall {\text{x}} \in {\text{U}}$, then

5 $${\tilde{\text{A}}} \cup {\tilde{\text{B}}} = \left\{ {\left( {{{\rm x}}, \max (\upmu _{{{\tilde{\text{A}}} }} \left( {\text{x}} \right),\upmu _{{{\tilde{\text{B}}} }} \left( {\text{x}} \right) } \right),\min \left( {\upeta _{{{\tilde{\text{A}}} }} \left( {\text{x}} \right),\upeta_{{{\tilde{\text{B}}} }} \left( {\text{x}} \right)} \right),\min \left( {\upupsilon _{{{\tilde{\text{A}}} }} \left( {\text{x}} \right),\upupsilon _{{{\tilde{\text{B}}} }} \left( {\text{x}} \right)} \right))|{\text{x}} \in {\text{U}}} \right\}$$

6 $${\tilde{\text{A}}} \cap {\tilde{\text{B}}} = \left\{ {\left( {{{\rm x}}, \min (\upmu _{{{\tilde{\text{A}}} }} \left( {\text{x}} \right),\upmu _{{{\tilde{\text{B}}} }} \left( {\text{x}} \right) } \right),\min \left( {\upeta _{{{\tilde{\text{A}}} }} \left( {\text{x}} \right),\upeta_{{{\tilde{\text{B}}} }} \left( {\text{x}} \right)} \right),\max \left( {\upupsilon _{{{\tilde{\text{A}}} }} \left( {\text{x}} \right),\upupsilon _{{{\tilde{\text{B}}} }} \left( {\text{x}} \right)} \right))|{\text{x}} \in {\text{U}}} \right\}$$

7 $${\tilde{\text{A}}} ^{{{\rm c}}} = \left\{ {x, \upupsilon_{{{\tilde{\text{A}}} }} \left( {\text{x}} \right), _{ } \upeta_{{{\tilde{\text{A}}} }} \left( {\text{x}} \right), \mu_{{{\tilde{\text{A}}} }} \left( {\text{x}} \right) |{\text{x}} \in {\text{U}}} \right\}$$

8 $$\tilde{A} \subseteq \tilde{B}\quad {\text{if}}\quad \left( {\mu_{{\tilde{A} }} \left( {\text{x}} \right) \le \mu_{{\tilde{B} }} \left( {\text{x}} \right), \upeta_{{\tilde{A} }} \left( {\text{x}} \right) \le \upeta_{{\tilde{B} }} \left( {\text{x}} \right), \upupsilon_{{\tilde{A} }} \left( {\text{x}} \right) \ge \upupsilon_{{\tilde{B} }} \left( {\text{x}} \right)\quad \forall {\text{x}} \in {\text{U}}} \right)$$

9 $$\tilde{A} = \tilde{B}\quad {\text{if}}\quad \tilde{A} \subseteq \tilde{B}\quad {\text{and}}\quad \tilde{B} \subseteq \tilde{A}$$

10 $$\tilde{A} \subseteq \tilde{B}\quad {\text{and}}\quad \tilde{B} \subseteq \tilde{C} \Rightarrow \tilde{A} \subseteq \tilde{C}$$

11 $$\left( {{\tilde{\text{A} }}^{{{\rm c}}} } \right)^{c} = {\tilde{\text{A} }}$$

### Operations

Let, ${\text{A}} = \left( {\mu_{{{\rm A}}} ,\upeta_{{{\rm A}}} ,\upupsilon_{{{\rm A}}} } \right)$ and ${\text{B}} = \left( {\mu_{{{\rm B}}} ,\upeta_{{{\rm B}}} ,\upupsilon_{{{\rm B}}} } \right)$ are any two PFNs. The following are some of the basic operations.

12 $${\text{A}} \oplus {\text{B}} = \left( {\mu_{{{\rm A}}} + \mu_{{{\rm B}}} - \mu_{{{\rm A}}} \mu_{{{\rm B}}} , \upeta_{{{\rm A}}} \upeta_{{{\rm B}}} , \upupsilon_{{{\rm A}}} \upupsilon_{{{\rm B}}} } \right)$$

13 $${\text{A}} \otimes {\text{B}} = \left( {\mu_{{{\rm A}}} \mu_{{{\rm B}}} , \upeta_{{{\rm A}}} + \upeta_{{{\rm B}}} - \upeta_{{{\rm A}}} \upeta_{{{\rm B}}} , \upupsilon_{{{\rm A}}} + \upupsilon_{{{\rm B}}} - \upupsilon_{{{\rm A}}} \upupsilon_{{{\rm B}}} } \right)$$

14 $$\uplambda {\text{A}} = \left( {1 - \left( {1 - \mu_{{{\rm A}}} } \right)^{\uplambda } ,\quad \upeta_{{{\rm A}}}^{\uplambda } , \upupsilon_{{{\rm A}}}^{\uplambda } } \right);\quad \uplambda > 0$$

15 $${\text{A}}^{\uplambda } = (\mu_{{{\rm A}}}^{\uplambda } , 1 - \left( {1 - \upeta_{{{\rm A}}} } \right)^{\uplambda } ,\quad 1 - \left( {1 - \upupsilon_{{{\rm A}}} } \right)^{\uplambda } ;\quad \uplambda > 0$$

16 $${\text{A}} \oplus {\text{B}} = {\text{B}} \oplus {\text{A}}$$

17 $${\text{A}} \otimes {\text{B}} = {\text{B}} \otimes {\text{A}}$$

18 $$\left( {{{\rm A}}^{\uplambda 1} } \right)^{\uplambda 2} = {\text{A}}^{\uplambda 1\uplambda 2}$$

19 $${\uplambda }\left( {{{\rm A}} \oplus {\text{B}}} \right) = {\uplambda A} \oplus {\uplambda B}$$

20 $$\left( {{{\rm A}} \otimes {\text{B}}} \right)^{\uplambda } = {\text{A}}^{\uplambda } \otimes {\text{B}}^{\uplambda }$$

### Defuzzification

The defuzzification of a PFN A is done in the following steps (Son 2017; Xu et al. 2019):

*Step 1*. Defining new positive and negative memberships

21 $$\mu_{{{\rm A}}}^{\prime } = \mu_{{{\rm A}}} + \frac{{\upeta_{{{\rm A}}} }}{2}$$

22 $$\upupsilon_{{{\rm A}}}^{\prime } = \upupsilon_{{{\rm A}}} + \frac{{\upeta_{{{\rm A}}} }}{2}$$

*Step 2*. Calculation of defuzzication value

23 $$\gamma_{{{\rm A}}} = \mu_{{{\rm A}}}^{\prime } + \pi_{{{\rm A }}} \left( {\frac{{1 + \mu_{{{\rm A}}}^{\prime } - \upupsilon_{{{\rm A}}}^{\prime } }}{2}} \right)$$

### Distance calculation

Two popular distance measures are defined in Cuong and Son (2015); Son 2016) as follows

Let, $\tilde{A} = {\text{x,}} \mu_{{\tilde{A} }} \left( {\text{x}} \right),\upeta_{{\tilde{A} }} \left( {\text{x}} \right),\upupsilon_{{\tilde{A} }} \left( {\text{x}} \right)$ and $\tilde{B} = {\text{x}}, \mu_{{\tilde{B} }} \left( {\text{x}} \right),\upeta_{{\tilde{B} }} \left( {\text{x}} \right),\upupsilon_{{\tilde{B} }} \left( {\text{x}} \right)$ are two PFS $\forall {\text{x}} \in {\text{U}}$ where ${\text{x}} = \left\{ {{{\rm x}}_{1} ,{\text{x}}_{2} ,{\text{x}}_{3} , \ldots .., {\text{x}}_{{{\rm n}}} } \right\}$.

Normalized Hamming distance:

24 $${\text{d}}^{{{\rm H}}} \left( {\tilde{A}, \tilde{B}} \right) = \frac{1}{{{\rm n}}} \mathop \sum \limits_{{{{\rm i}} = 1}}^{{{\rm n}}} \left( {\left| {\mu_{{\tilde{A} }} \left( {{{\rm x}}_{{{\rm i}}} } \right) - \mu_{{\tilde{B} }} \left( {{{\rm x}}_{{{\rm i}}} } \right) } \right| + \left| {\upeta_{{\tilde{A} }} \left( {{{\rm x}}_{{{\rm i}}} } \right) - \upeta_{{\tilde{B} }} \left( {{{\rm x}}_{{{\rm i}}} } \right) } \right| + \left| {\upupsilon_{{\tilde{A} }} \left( {{{\rm x}}_{{{\rm i}}} } \right) - \upupsilon_{{\tilde{B} }} \left( {{{\rm x}}_{{{\rm i}}} } \right) } \right|} \right)$$

Normalized Euclidean distance:

25 $${\text{d}}^{{{\rm E}}} \left( {\tilde{A},~\tilde{B}} \right) = \sqrt {\frac{1}{{{\rm n}}}~\mathop \sum \limits_{{{{\rm i}} = 1}}^{{{\rm n}}} \left( {\left( {\mu _{{\tilde{A}~}} \left( {{{\rm x}}_{{{\rm i}}} } \right) - \mu _{{\tilde{B}~}} \left( {{{\rm x}}_{{{\rm i}}} } \right)} \right)^{2} + \left( {\upeta _{{\tilde{A}~}} \left( {{{\rm x}}_{{{\rm i}}} } \right) - \upeta _{{\tilde{B}~}} \left( {{{\rm x}}_{{{\rm i}}} } \right)} \right)^{2} + \left( {\upupsilon _{{\tilde{A}~}} \left( {{{\rm x}}_{{{\rm i}}} } \right) - \upupsilon _{{\tilde{B}~}} \left( {{{\rm x}}_{{{\rm i}}} } \right)} \right)^{2} } \right)}$$

### Score and accuracy functions

The score function of any PFN A is given as (Cuong and Kreinovich 2013)

26 $${\text{S}}_{{{\rm A}}} = \mu_{{{\rm A}}} - \upupsilon_{{{\rm A}}}$$

The accuracy function is defined as

27 $${\text{H}}_{{{\rm A}}} = \mu_{{{\rm A}}} + \upeta_{{{\rm A}}} + \upupsilon_{{{\rm A}}}$$

In this regard, the rules for comparing any two PFNs such as A and B are given below

(i) $${\text{If\,\,\,S}}_{{{\rm A}}} \prec {\text{S}}_{{{\rm B}}} ,\quad {\text{then\,\,\,A}} \prec {\text{B}}$$
(ii) $${\text{If\,\,\,S}}_{{\rm A}} \succ {\text{S}}_{{{\rm B}}} ,\quad {\text{then\,\,\,A}} \succ {\text{B}}$$
(iii) $${\text{If\,\,\,S}}_{{\rm A}} = {\text{S}}_{{\rm B}} ,\quad {\text{H}}_{{\rm A}} \prec {\text{H}}_{{\rm B}} ,\quad {\text{then\,\,\,A}} \prec {\text{B}}$$
(iv) $${\text{If\,\,\,S}}_{{\rm A}} = {\text{S}}_{{\rm B}} ,{\text{H}}_{{\rm A}} \succ {\text{H}}_{{\rm B}} ,\quad {\text{then\,\,\,\,A}} \succ {\text{B}}$$
(v) $${\text{If\,\,\,S}}_{{\rm A}} = {\text{S}}_{B} ,\quad {\text{H}}_{{\rm A}} = {\text{H}}_{{{\rm B}}} ,\quad {\text{then\,\,\,\,A}} = {\text{B}}$$

However, Si et al. (Si et al. 2019) reviewed the calculation of score values using above-mentioned conventional definitions and proposed a modified version. They proposed definitions of absolute and actual scores using all three membership functions such as positive, neutral and negative.

### Absolute and actual score

The steps for calculation are described below (Si et al. 2019)

*Step 1*. Identification of the positive ideal solution (PIS).

For a set of n number of PFNs, PIS is given as

28 $${\text{Z}}^{ + } = \left( {\mu^{ + } , \upeta^{ + } , \upupsilon^{ + } } \right) = (\mathop {{{\rm max}}}\limits_{{{\rm i}}} \,\mu_{i} , \mathop {{{\rm min}}}\limits_{{{\rm i}}} \,\upeta_{i} , \mathop {{{\rm min}}}\limits_{{{\rm i}}} \,\upupsilon_{i} ),\quad {\text{where\,\,\,i}} = 1,2, \ldots {\text{n}}$$

*Step 2*. Find out goal differences for each PFN

29 $${\text{Positivegoaldifference:}}\quad\upmu _{{{{\rm i}} + }} = \mu^{ + } - \mu_{{{\rm i}}}$$

30 $${\text{Negative\,goal\,difference:}}\,\upupsilon_{{{{\rm i}} - }} = \upupsilon_{{{\rm i}}} - \upupsilon^{ + }$$

*Step 3*. Find out the average neutral degree

31 $$\overline{\upeta } = \frac{1}{{{\rm n}}} \mathop \sum \limits_{{{{\rm i}} = 1}}^{{{\rm n}}} \upeta_{{{\rm i}}}$$

*Step 4*. Calculation of the absolute score for each PFN

32 $${\text{S}}_{{{{\rm i}}\left( {{{\rm abs}}} \right)}} = \left( {1 - \mu_{{{{\rm i}} + }} } \right) - \upupsilon_{{{{\rm i}} - }}$$

*Step 5*. Derive the actual score for each PFN

33 $${\text{S}}_{{{{\rm i}}\left( {{{\rm act}}} \right)}} = \frac{{{{\rm S}}_{{{{\rm i}}\left( {{{\rm abs}}} \right)}} }}{{1 - \left( {\overline{\upeta } - \upeta_{{{\rm i}}} } \right)}}$$

Here, the following rules are applicable

$${\text{If\,\,\,S}}_{{{{\rm A}}\left( {{{\rm act}}} \right)}} \succ {\text{S}}_{{{{\rm B}}\left( {{{\rm act}}} \right)}} \quad {\text{then\,\,\,A}} \succ {\text{B}}$$

$${\text{If\,\,\,S}}_{{{{\rm A}}\left( {{{\rm act}}} \right)}} = {\text{S}}_{{{{\rm B}}\left( {{{\rm act}}} \right)}} \quad {\text{then\,if}}\quad \mu_{{{\rm A}}} > \mu_{{{\rm B}}} \quad {\text{and}}\quad \upeta_{{{\rm A}}} \ge \upeta_{{{\rm B}}} \quad {\text{then\,\,\,A}} \succ {\text{B }}$$

$${\text{If\,\,\,S}}_{{{{\rm A}}\left( {{{\rm act}}} \right)}} = {\text{S}}_{{{{\rm B}}\left( {{{\rm act}}} \right)}} \quad {\text{and}}\quad \mu_{{{\rm A}}} \ge \mu_{{{\rm B}}} \quad {\text{and}}\quad \upeta_{{{\rm A}}} < \upeta_{{{\rm B}}} \quad {\text{then\,if}}\quad \upupsilon_{{{\rm A}}} \le \upupsilon_{{{\rm B}}} \quad {\text{then\,\,\,A}} \succ {\text{B}},\quad {\text{otherwise\,A}} \prec {\text{B}}$$

As $$\left( {\overline{\upeta } - \upeta_{{{\rm i}}} } \right) \ne 1, {\text{S}}_{{{\rm i}\left( {{{\rm act}}} \right)}} \,{\text{is\,always\,finite}}.$$

## EDAS method

The computational steps of the original EDAS method (Ghorabaee et al. 2015) are given below.

*Step 1*: Formulation of the decision-making matrix (X) given as:

X = [X_ij_]_m×n_; where X_ij_: Performance value of ith alternative on jth criterion.

*Step 2:* Calculation of the average solution

34 $${\text{AV}}_{{\rm j}} = \frac{{\mathop \sum \nolimits_{{{{\rm i}} = 1}}^{{{\rm m}}} {\text{x}}_{{{\rm ij}}} }}{{{\rm m}}};\quad {\text{j}} = 1,2, \ldots .{\text{n}}$$

*Step 3*: Calculation of PDA and NDAIf jth criterion is beneficial,

$$\begin{aligned} {\text{PDA}} & = \left[ {{{\rm PDA}}_{{{{\rm ij}}}} } \right]_{{{{\rm m}} \times {\text{n}}}} , \\ {\text{NDA}} & = \left[ {{{\rm NDA}}_{{{{\rm ij}}}} } \right]_{{{{\rm m}} \times {\text{n}}}} \\ \end{aligned}$$

35 $${\text{PDA}}_{{{{\rm ij}}}} = \frac{{\max \left( {0, \left( {{{\rm x}}_{{{{\rm ij}}}} - {\text{ AV}}_{{{\rm j}}} } \right)} \right)}}{{{{\rm AV}}_{{{\rm j}}} }}$$

36 $${\text{NDA}}_{{{{\rm ij}}}} = \frac{{\max \left( {0, \left( {{{\rm AV}}_{{{\rm j}}} - {\text{x}}_{{{{\rm ij}}}} } \right)} \right)}}{{{{\rm AV}}_{{{\rm j}}} }}$$

If jth criterion is non-beneficial,

37 $${\text{PDA}}_{{{{\rm ij}}}} = \frac{{\max \left( {0, \left( {{{\rm AV}}_{{{\rm j}}} - {\text{x}}_{{{{\rm ij}}}} } \right)} \right)}}{{{{\rm AV}}_{{{\rm j}}} }}$$

38 $${\text{NDA}}_{{{{\rm ij}}}} = \frac{{\max \left( {0, \left( {{{\rm x}}_{{{{\rm ij}}}} - {\text{AV}}_{{{\rm j}}} } \right)} \right)}}{{{{\rm AV}}_{{{\rm j}}} }}$$

*Step 4*: Determine the weighted sum of PDA and NDA for all alternatives

39 $${\text{SP}}_{{{\rm i}}} = \mathop \sum \limits_{{{{\rm j}} = 1}}^{{{\rm n}}} {\text{w}}_{{{\rm j }}} {\text{PDA}}_{{{\rm ij }}}$$

40 $${\text{SN}}_{{{\rm i}}} = \mathop \sum \limits_{{{{\rm j}} = 1}}^{{{\rm n}}} {\text{w}}_{{{\rm j }}} {\text{NDA}}_{{{\rm ij }}}$$

where w_j_ is the weight of jth criterion.

*Step 5:* Normalization of the values of SP and SN for all the alternatives

41 $${\text{NSP}}_{i} = \frac{{{{\rm SP}}_{{{\rm i}}} }}{{\mathop {{{\rm max}}}\limits_{{{\rm i}}} \left( {{{\rm SP}}_{{{\rm i}}} } \right)}}$$

42 $${\text{NSN}}_{{{\rm i}}} = 1 - \frac{{{{\rm SN}}_{{{\rm i}}} }}{{\mathop {{{\rm max}}}\limits_{{{\rm i}}} \left( {{{\rm SN}}_{{{\rm i}}} } \right)}}$$

*Step 6:* Calculation of the appraisal score (AS) for all alternatives

43 $${\text{AS}}_{{{\rm i}}} = \frac{1}{2} \left( {{{\rm NSP}}_{{{\rm i}}} + {\text{NSN}}_{{{\rm i}}} } \right)$$

where 0 ≤ AS_i_ ≤ 1. The alternative having the highest AS_i_ is ranked first and so on.

## Proposed methodology: grey correlational picture fuzzy EDAS (GCPF-EDAS)

In this section we present the computational steps of the proposed GCPF-EDAS methodology for multi-criteria based group decision-making extending the work of Zhang et al. (2019); Das and Chakraborty 2022) based on the original algorithm of EDAS (Ghorabaee et al. 2015).

Suppose,

M_i_, where ${\text{i}} = 1,2, \ldots {\text{m}} \left( {{{\rm m\,is\,finite\,and}} \ge 2} \right)$ are the number of alternatives under comparison subject to a set of attributes or criteria, C_j_, where ${\text{j}} = 1,2, \ldots {\text{n}} \left( {{{\rm n\,is\,finite\,and}}\, \ge 2} \right)$, based on the opinions of DM_k_, where ${\text{k}} = 1,2, \ldots {\text{r }}\left( {{{\rm r\,is\,finite\,and}}\, \ge 2} \right)$ are the number of decision-makers.

The steps of the proposed algorithm are as follows.

*Step 1*. Construction of the linguistic weight matrix for the attributes

44 $$\varphi^{{{\rm k}}} = \begin{array}{*{20}c} {\begin{array}{*{20}c} {{{\rm C}}_{1} } \\ {{{\rm C}}_{2} } \\ \cdot \\ \end{array} } \\ \cdot \\ {{{\rm C}}_{{{\rm n}}} } \\ \end{array} \left[ {\begin{array}{*{20}c} {\varphi_{1}^{{{\rm k}}} } \\ {\varphi_{2}^{{{\rm k}}} } \\ {\begin{array}{*{20}c} \cdot \\ \cdot \\ {\varphi_{{{\rm n}}}^{{{\rm k}}} } \\ \end{array} } \\ \end{array} } \right]$$

Here, $\varphi_{{{\rm j}}}^{{{\rm k}}}$ is the relative importance (in linguistic scale) given by DM_k_ (where, ${\text{k}} = 1,2, \ldots {\text{r}}$) for each criterion C_j_ (where, ${\text{j}} = 1,2, \ldots {\text{n }}$). The DMs express their views as positive, neutral, and negative and may refuse to give opinions as well. In this research, for finding out relative importance of the criteria, we do not allow the DMs to refuse as the criteria used for a common real-life problem are sufficiently known to all. Therefore, we offer the DMs to select any of the following three categories for their response with respect to each criterion (see Table 1).

**Table 1:** Linguistic scale for weighting the criteria

Notation	Meaning and/or indication
H	High importance (positive membership)
N	neither high nor low importance (neutral membership)
L	low importance (negative membership)

*Step 2.* Formulation of the PF criteria weight matrix.

The criteria matrix is represented as ${\text{C}}_{{{\rm w}}} = \left[ {{{\rm C}}_{{{{\rm wj}}}} } \right]_{{{{\rm n}} \times 1}}$.

Here, ${\text{C}}_{{{{\rm wj}}}} = \mu_{{{\rm j}}} ,\upeta_{{{\rm j}}} ,\upupsilon_{{{\rm j}}}$ is a PFN showing the relative importance of the criterion C_j_ considering the responses of all DMs. The aggregation of the individual DM’s response can be done in several ways. We refer the method followed in Jovčić et al. (2020) and accordingly, the PFNs corresponding to the criteria are calculated in terms of the proportion of type of responses (positive, neutral, and negative) opined by the DMs.

*Step 3*. Calculation of criteria weights

The weight for the criterion C_j_ is given as (Jovčić et al. 2020)

45 $${\text{w}}_{{{\rm j}}} = \frac{{\mu_{{{\rm j}}} + \frac{{\upeta_{{{\rm j}}} }}{2} + \frac{1}{2}\pi_{{{\rm j}}} \left( {1 + \mu_{{{\rm j}}} - \upupsilon_{{{\rm j}}} } \right)}}{{\mathop \sum \nolimits_{{{{\rm j}} = 1}}^{{{\rm n}}} \left[ {\mu_{{{\rm j}}} + \frac{{\upeta_{{{\rm j}}} }}{2} + \frac{1}{2}\pi_{{{\rm j}}} \left( {1 + \mu_{{{\rm j}}} - \upupsilon_{{{\rm j}}} } \right)} \right]}},\quad {\text{j}} = 1,2,..{\text{n}};\quad {\text{w}}_{{{\rm j}}} \in \left[ {0,1} \right]\quad {\text{and}}\quad \mathop \sum \limits_{{{{\rm j}} = 1}}^{{{\rm n}}} {\text{w}}_{{{\rm j}}} = 1$$

Here, π_j_ represents the degree of refusal (refer the expression (3)).

*Step 4*. Formulation of the linguistic evaluation matrix.

The linguistic evaluation matrix (for individual responses) is given by

46 $$\Psi = \left[ {\zeta_{{{{\rm ij}}}}^{{{\rm k}}} } \right]_{{{{\rm m}} \times {\text{n}}}} ;\quad {\text{k}} = 1,2, \ldots {\text{r}}$$

$\zeta_{{{{\rm ij}}}}^{{{\rm k}}}$ is the linguistic evaluation expressed by kth DM for ith alternative with respect to jth criterion. Again, in general, a respondent may express positive, neutral, negative or refusal opinion respectively.

*Step 5*. Formulation of the PF-evaluation matrix.

The PF-evaluation matrix is represented as

47 $$\Gamma = \left[ {\tau_{{{{\rm ij}}}} } \right]_{{{{\rm m}} \times {\text{n}}}}$$

Here, $\tau_{{{{\rm ij}}}} = \mu_{{{{\rm ij}}}} ,\upeta_{{{{\rm ij}}}} ,\upupsilon_{{{{\rm ij}}}}$ represents a PFN for evaluation of the ith alternative with respect to the jth criterion by the DMs. The PFNs are calculated in terms of the proportion of type of responses (positive, neutral, and negative) opined by the DMs.

*Step 6.* Determination of PF-decision matrix.

The PF-decision matrix is formed after normalization of the PF-evaluation matrix.

48 $$\Omega = \left[ {\gamma_{{{{\rm ij}}}} } \right]_{{{{\rm m}} \times {\text{n}}}}$$

where,

49 $$\gamma_{ij} = \left\{ {\begin{array}{*{20}l} {\tau_{ij} = \mu_{ij} ,\upeta_{ij} ,\upupsilon_{ij} ;} \hfill & {\quad {\text{for}}{\mkern 1mu} {\text{beneficial}}{\mkern 1mu} {\text{criteria}}} \hfill \\ {\tau_{ij}^{c} = \upupsilon_{ij} , \upeta_{ij} , \mu_{ij} ;} \hfill & {\quad {\text{for}}{\mkern 1mu} {\text{non - beneficial}}{\mkern 1mu} {\text{criteria}}} \hfill \\ \end{array} } \right.$$

*Step 7*. Computation of the average solution

50 $$\overline{\gamma }_{{{\rm j}}} = \left[ {\frac{{\mathop \sum \nolimits_{{{{\rm i}} = 1}}^{{{\rm m}}} \gamma_{{{{\rm ij}}}} }}{{{\rm m}}}} \right]_{{1 \times {\text{n}}}}$$

$\overline{\gamma }_{{{\rm j}}}$ is also a PFN ${\upmu }_{{{{\rm ij}}}}^{{\prime }} ,{\upeta }_{{{{\rm ij}}}}^{{\prime }} ,{\upupsilon }_{{{{\rm ij}}}}^{{\prime }} { }$ wherein the membership functions are average of the membership values of γ_ij_ for each criterion. For example, $\mu_{ij}^{{\prime }} = \left[ {\frac{{\mathop \sum \nolimits_{{{{\rm i}} = 1}}^{{{\rm m}}} \mu_{{{{\rm ij}}}} }}{{{\rm m}}}} \right]_{{1 \times {\text{n}}}}$.

*Step 8*. Derive the positive distance from average (PDA) and negative distance from average (NDA)

51 $${\text{PDA:}}\quad \left[ {{{\rm D}}_{{{{\rm ij}}}}^{ + } } \right]_{{{{\rm m}} \times {\text{n}}}} = \frac{{{{\rm Max}} \left( {0, \left( {{{\rm S}}\left( {\gamma_{{{{\rm ij}}}} } \right) - {\text{S}}\left( {\overline{\gamma }_{{{\rm j}}} } \right)} \right)} \right)}}{{{{\rm S}}\left( {\overline{\gamma }_{{{\rm j}}} } \right)}}$$

52 $${\text{NDA:}}\quad \left[ {{{\rm D}}_{{{{\rm ij}}}}^{ - } } \right]_{{{{\rm m}} \times {\text{n}}}} = \frac{{{{\rm Max}} \left( {0, \left( {{{\rm S}}\left( {\overline{\gamma }_{{{\rm j}}} } \right) - {\text{S}}\left( {\gamma_{{{{\rm ij}}}} } \right)} \right)} \right)}}{{{{\rm S}}\left( {\overline{\gamma }_{{{\rm j}}} } \right)}}$$

where the score value is calculated using the conventional way (refer the Eq. (26)).

*Step 9*. Calculation of the grey correlation coefficient (GC) values

53 $${\text{GP}}_{{{{\rm ij}}}} = \frac{{{{\rm D}}_{{{{\rm ijmin}}}}^{ + } + \xi {\text{D}}_{{{{\rm ijmax}}}}^{ + } }}{{\left| {{{\rm D}}_{{{{\rm ijmax}}}}^{ + } - {\text{D}}_{{{{\rm ij}}}}^{ + } } \right| + \xi {\text{D}}_{{{{\rm ijmax}}}}^{ + } }}$$

54 $${\text{GN}}_{{{{\rm ij}}}} = \frac{{{{\rm D}}_{{{{\rm ij\,min}}}}^{ - } + \xi {\text{D}}_{{{{\rm ij\,max}}}}^{ - } }}{{\left| {{{\rm D}}_{{i{\text{j\,max}}}}^{ - } - {\text{D}}_{{{{\rm ij}}}}^{ - } } \right| + \xi {\text{D}}_{{{{\rm ij\,max}}}}^{ - } }}$$

Here, ξ is the differentiating or distinguishing or identification coefficient whose value is usually taken as 0.5 as suggested in Das and Chakraborty (2022) which is a neutral position.

*Step 10.* Calculation of the average weighted GC values

55 $${\text{GCP}}_{{{\rm i}}} = \frac{1}{{{\rm n}}} \mathop \sum \limits_{{{{\rm j}} = 1}}^{{{\rm n}}} {\text{w}}_{{{\rm j}}} {\text{GP}}_{{{{\rm ij}}}} { }$$

56 $${\text{GCN}}_{{{\rm i}}} = \frac{1}{{{\rm n}}} \mathop \sum \limits_{{{{\rm j}} = 1}}^{{{\rm n}}} {\text{w}}_{{{\rm j}}} {\text{GN}}_{{{{\rm ij}}}} { }$$

*Step 11*. Normalization of the average weighted GC values

57 $${\text{N-GCP:\,GCP}}_{{{\rm i}}}^{{\prime }} = \frac{{{{\rm GCP}}_{{{\rm i}}} }}{{\mathop {{{\rm max}}}\limits_{{{\rm i}}} {\text{GCP}}_{{{\rm i}}} }}$$

58 $${\text{N-GCN:\,GCN}}_{i}^{\prime } = 1 - \frac{{{{\rm GCN}}_{{{\rm i}}} }}{{\mathop {{{\rm max}}}\limits_{{{\rm i}}} {\text{GCN}}_{{{\rm i}}} }}$$

*Step 12*. Calculation of the grey-based appraisal score

59 $${\text{GAS}}_{{{\rm i}}} = \frac{1}{2}\left( {{{\rm GCP}}_{i}^{{\prime }} + {\text{GCN}}_{{{\rm i}}}^{{\prime }} } \right)$$

Here, $0 \le {\text{GAS}}_{{{\rm i}}} \le 1$; the decision rule is: *Higher the value, more is the preference.*

## Case study: M-Wallet selection

In this paper, we deal with the problem of selection of mobile wallet based on user based views. We select a list of widely used mobile wallets in India. These wallets have been introduced in the market by various public and private bodies for multiple uses. Table 2 provides the list of wallets under comparison in this study.

**Table 2:** List of mobile wallets

Mobile wallets	Code	Mobile wallets	Code
PayTM	A1	JioMoney	A8
Google Pay	A2	HDFC PayZapp	A9
PhonePe	A3	Mobikwik	A10
Amazon Pay	A4	YonoSBI	A11
Freecharge	A5	Airtel Payment Banks	A12
BHIM Axis Pay	A6	PayU	A13
ICICI Pockets	A7	Citi MasterPass	A14

We then move to nominate a group of users for multi-criteria group decision based analysis. For selection of respondents or DMs, we consider the aspects like varying age group, employment, qualification, and the frequency of usage of M-Wallets (which is an indicator of familiarity of use and awareness. we considered those who use mobile wallets at a reasonably high frequency). Accordingly, in the present study 10 respondents have participated. Therefore, in this paper, the number of DMs is 10 (r = 10) which satisfies the condition for sample size to be used in a typical group-decision making set up (Kendall 1948; Turskis et al. 2019; Biswas 2020).

The next step is the selection of criteria. We follow the findings of the past work in line with the theoretical framework of TAM and UTAUT to select the criteria. The descriptions of the criteria are given in Table 3.

**Table 3:** List of criteria

Criteria	Code	Description	Effect direction
Features	C1	Variety of applications, options	( +)
Convenience to use	C2	Easy to use anywhere, anytime, user friendly	( +)
Compatibility	C3	Accessible on any device and any operating system	( +)
Speed	C4	Time taken to complete a transaction	( +)
Offers and Discounts		Cash back offers, vouchers	( +)
Security and Privacy	C6	Information security	( +)
Transaction cost	C7	processing charge	( −)

Therefore, in this study, i = 14; j = 7 and r = 10. In the following steps we present the detailed analysis.

*Step 1*. The linguistic weight matrix for the attributes or criteria is given in Table 4. The respondents rate the criteria according to their relative priority using the scale given in the Table 1.

**Table 4:** Linguistic evaluation of the criteria by the DMs

Criteria	DMs									
	DM1	DM2	DM3	DM4	DM5	DM6	DM7	DM8	DM9	DM10
C1	H	H	H	N	H	N	H	N	H	N
C2	H	H	H	H	H	H	H	H	H	H
C3	N	H	N	N	H	H	N	L	H	H
C4	N	H	H	H	H	H	H	N	H	H
C5	H	H	H	H	H	N	L	H	N	N
C6	H	N	H	H	H	H	N	H	H	N
C7	N	N	L	H	L	H	L	L	H	N

*Step 2*. Using the methodology explained earlier, we then formulate the PF criteria weight matrix (see Table 5). Since, during the evaluation of the relative importance of criteria, the DMs are not allowed to refuse, μ + η + ν = 1.0

**Table 5:** PF criteria matrix for weight calculation

Criteria	μ	η	ν
C1	0.6	0.4	0.0
C2	1.0	0.0	0.0
C3	0.5	0.4	0.1
C4	0.8	0.2	0.0
C5	0.6	0.3	0.1
C6	0.7	0.3	0.0
C7	0.3	0.3	0.4

*Step 3*. Using the expression (45), in this step we calculate the criteria weights. Since, the option for refuse is not present in this phase, π_j_ = 0. Accordingly, the weights are calculated (refer Table 6). For example, ${\text{w}}_{1} = \frac{{0.6 + \frac{1}{2} \times 0.4}}{5.5} = 0.1468;{\text{w}}_{2} = \frac{{1.0 + \frac{1}{2} \times 0.0}}{5.5} = 0.1835$.

**Table 6:** Criteria weights

Criteria		μ	η	ν	μ + η/2	Weight
C1	( +)	0.6	0.4	0.0	0.8	0.1468
C2	( +)	1.0	0.0	0.0	1.0	0.1835
C3	( +)	0.5	0.4	0.1	0.7	0.1284
C4	( +)	0.8	0.2	0.0	0.9	0.1651
C5	( +)	0.6	0.3	0.1	0.8	0.1376
C6	( +)	0.7	0.3	0.0	0.9	0.1560
C7	( −)	0.3	0.3	0.4	0.5	0.0826
				Sum	5.5	

*Step 4*. Now we proceed for linguistic evaluation of the alternatives (i.e., mobile wallets) with respect to the criteria used here. For such purpose, now the respondents are allowed to refuse also, as we assume all DMs do not use all wallets. Hence, for some criteria, the DMs may not be able to rate all alternatives appropriately. As we see, C7 is the cost element. Therefore, for C7 we prefer to use high, low, and neither high nor low options whereas, for all other criteria we use good, bad, and neither good nor bad ratings. Table 7 exhibits the linguistic evaluation scales and Table 8 shows the rating of all alternatives with respect to the criteria as opined by the DMs. Here, ‘R’ stands for refusal.

**Table 7:** Linguistic evaluation scale for the alternatives

Notion	Meaning	Indication
G	Good	Positive membership
P	Poor	Negative membership
H	High	Positive membership
L	Low	Negative membership
N	Neither high nor low/ neither good nor poor	Neutral membership

**Table 8:** Linguistic evaluation of the alternatives

Criteria	DMs	A1	A2	A3	A4	A5	A6	A7	A8	A9	A10	A11	A12	A13	A14
C1	DM1	G	G	G	R	R	R	R	N	N	N	G	N	N	N
	DM2	P	G	G	G	R	R	R	R	P	P	P	G	R	R
	DM3	G	N	G	P	N	P	R	N	R	N	P	N	G	R
	DM4	N	N	G	G	R	P	R	R	N	P	G	R	R	R
	DM5	G	N	G	N	R	R	R	R	R	R	R	R	R	R
	DM6	N	G	G	P	P	G	P	N	P	P	P	P	P	P
	DM7	R	R	G	R	R	R	R	R	R	R	R	R	R	R
	DM8	G	N	N	N	R	P	R	P	R	N	R	N	N	P
	DM9	G	R	G	G	R	R	R	R	R	R	G	N	R	R
	DM10	N	G	G	N	R	G	N	N	N	N	G	G	N	R
C2	DM1	G	G	G	R	R	R	R	N	N	N	G	N	N	N
	DM2	P	G	G	G	R	R	R	R	P	R	P	R	R	R
	DM3	G	G	G	G	N	P	R	N	R	N	N	N	G	R
	DM4	G	G	G	G	N	P	P	P	G	P	G	G	R	R
	DM5	G	N	G	G	R	R	R	R	R	R	R	R	R	R
	DM6	G	G	G	R	R	N	R	R	N	R	R	R	R	R
	DM7	R	R	G	R	R	R	R	R	R	R	R	R	R	R
	DM8	G	G	N	G	R	P	R	N	R	P	R	G	N	P
	DM9	G	R	G	G	R	R	R	R	R	R	G	N	R	R
	DM10	N	G	G	N	N	G	G	G	G	N	G	G	G	N
C3	DM1	G	G	G	N	N	N	R	G	R	R	G	R	R	R
	DM2	G	G	G	G	R	R	R	R	R	R	G	G	R	R
	DM3	G	G	G	G	N	P	R	G	R	N	P	N	G	R
	DM4	G	G	G	G	N	P	P	P	N	R	G	G	R	R
	DM5	G	P	G	G	R	R	R	R	R	R	R	R	R	R
	DM6	G	G	G	N	N	G	R	R	R	R	R	R	R	R
	DM7	R	R	G	R	R	R	R	R	R	R	R	R	R	R
	DM8	N	G	N	G	R	P	R	N	R	N	R	N	N	P
	DM9	G	R	G	G	R	R	R	R	R	R	G	R	R	R
	DM10	N	G	G	G	N	N	N	N	G	N	G	G	G	G
C4	DM1	N	G	G	N	N	R	R	N	R	R	P	R	R	R
	DM2	R	G	G	G	R	P	R	R	R	R	R	R	R	R
	DM3	N	G	G	G	N	N	R	N	R	N	R	N	N	R
	DM4	N	G	G	G	P	P	P	P	G	N	N	N	R	R
	DM5	G	G	G	G	R	R	R	R	R	R	R	R	R	R
	DM6	G	G	G	N	P	G	R	R	N	R	R	R	R	R
	DM7	R	R	G	R	R	R	R	R	R	R	R	R	R	R
	DM8	G	G	G	N	R	P	R	N	R	N	R	G	N	P
	DM9	N	R	N	G	R	R	R	R	R	R	N	R	R	R
	DM10	N	G	G	G	N	N	G	N	G	N	G	G	G	N
C5	DM1	G	P	G	N	N	R	R	N	R	R	P	R	R	R
	DM2	R	P	G	R	R	R	R	R	R	R	R	R	R	R
	DM3	G	N	G	G	N	P	R	G	R	N	P	N	N	R
	DM4	G	G	G	G	R	P	P	P	G	P	P	P	P	P
	DM5	G	G	G	G	R	R	R	R	R	R	R	R	R	R
	DM6	G	G	G	G	P	P	R	R	R	R	R	R	R	R
	DM7	R	R	G	R	R	R	R	R	R	R	R	P	R	R
	DM8	G	N	N	G	R	P	R	N	R	N	R	P	N	N
	DM9	N	R	N	G	R	R	R	R	R	R	R	R	R	R
	DM10	N	G	G	G	N	N	G	N	G	N	G	G	N	G
C6	DM1	G	G	G	R	R	R	R	N	R	R	G	R	R	R
	DM2	P	G	G	G	R	R	R	R	G	R	G	G	R	R
	DM3	N	G	N	N	N	G	R	N	R	N	G	N	N	R
	DM4	G	G	G	G	R	P	R	R	G	R	R	R	R	R
	DM5	G	G	G	G	R	R	R	R	R	R	R	R	R	R
	DM6	G	G	G	G	P	G	R	R	G	R	R	R	R	R
	DM7	R	R	G	R	R	R	R	R	R	R	R	R	R	R
	DM8	P	N	N	N	R	G	R	P	R	N	R	N	P	G
	DM9	N	R	N	N	R	R	R	R	R	R	G	R	R	R
	DM10	N	G	G	N	N	N	G	N	G	N	G	G	G	G
C7	DM1	L	L	L	H	R	R	R	R	R	R	H	R	R	R
	DM2	L	L	L	L	H	R	R	R	L	R	L	L	R	R
	DM3	L	L	L	R	N	H	R	N	R	N	H	N	N	R
	DM4	H	L	L	L	H	H	N	L	R	H	L	L	R	R
	DM5	H	H	H	H	R	R	R	R	R	R	R	R	R	R
	DM6	N	N	N	N	R	R	R	R	R	R	R	R	R	R
	DM7	R	R	H	R	R	R	R	R	R	R	R	R	R	R
	DM8	L	L	L	N	R	H	R	N	R	N	R	N	L	H
	DM9	L	R	L	L	R	R	R	R	R	R	L	R	R	R
	DM10	N	H	H	N	N	H	H	N	H	H	H	H	H	H

*Step 5.* In this step we form the PF-evaluation matrix based on the responses of the DMs (refer Tables 7 and 8). We find the degrees of positive, neutral and negative memberships by proportionate responses (Good or High; Neither Good nor Low or Neither High nor Low; Poor or Bad) and find out the respective PFNs. For example,

For the alternative A1 subject to the influence of the criterion C1:

Good = 5 responses; Poor = 1 response; Neither Good nor Poor = 3 responses; Refusal = 1 response.

$$\Rightarrow \mu = 0.5; \upeta = 0.3; \upupsilon = 0.1$$

Likewise, we calculate all other PFNs to construct the matrix $\Gamma = \left[ {\tau_{{{{\rm ij}}}} } \right]_{{{{\rm m}} \times {\text{n}}}}$ as shown in the Table 9.

**Table 9:** PF evaluation matrix

Mobile wallets	Evaluating criteria																				
	C1			C2			C3			C4			C5			C6			C7		
	( +)			( +)			( +)			( +)			( +)			( +)			( −)		
	μ	η	ν	μ	η	ν	μ	η	ν	μ	η	ν	μ	η	ν	μ	η	ν	μ	η	ν
A1	0.5	0.3	0.1	0.7	0.1	0.1	0.7	0.2	0.0	0.3	0.5	0.0	0.6	0.2	0.0	0.4	0.3	0.2	0.2	0.2	0.5
A2	0.4	0.4	0.0	0.7	0.1	0.0	0.7	0.0	0.1	0.8	0.0	0.0	0.4	0.2	0.2	0.7	0.1	0.0	0.2	0.1	0.5
A3	0.9	0.1	0.0	0.9	0.1	0.0	0.9	0.1	0.0	0.9	0.1	0.0	0.8	0.2	0.0	0.7	0.3	0.0	0.3	0.1	0.6
A4	0.3	0.3	0.2	0.6	0.1	0.0	0.7	0.2	0.0	0.6	0.3	0.0	0.7	0.1	0.0	0.4	0.4	0.0	0.2	0.3	0.3
A5	0.0	0.1	0.1	0.0	0.3	0.0	0.0	0.5	0.0	0.0	0.3	0.2	0.0	0.3	0.1	0.0	0.2	0.1	0.2	0.2	0.0
A6	0.2	0.0	0.3	0.1	0.1	0.3	0.1	0.2	0.3	0.1	0.2	0.3	0.0	0.1	0.4	0.3	0.1	0.1	0.4	0.0	0.0
A7	0.0	0.1	0.1	0.1	0.0	0.1	0.0	0.1	0.1	0.1	0.0	0.1	0.1	0.0	0.1	0.1	0.0	0.0	0.1	0.1	0.0
A8	0.0	0.4	0.1	0.1	0.3	0.1	0.2	0.2	0.1	0.0	0.4	0.1	0.1	0.3	0.1	0.0	0.3	0.1	0.0	0.3	0.1
A9	0.0	0.3	0.2	0.2	0.2	0.1	0.1	0.1	0.0	0.2	0.1	0.0	0.2	0.0	0.0	0.4	0.0	0.0	0.1	0.0	0.1
A10	0.0	0.4	0.3	0.3	0.0	0.2	0.0	0.3	0.0	0.0	0.4	0.0	0.0	0.3	0.1	0.0	0.3	0.0	0.2	0.2	0.0
A11	0.4	0.0	0.3	0.4	0.1	0.1	0.5	0.0	0.1	0.1	0.2	0.1	0.1	0.0	0.3	0.5	0.0	0.0	0.3	0.0	0.3
A12	0.2	0.4	0.1	0.3	0.3	0.0	0.3	0.2	0.0	0.2	0.2	0.0	0.1	0.1	0.3	0.2	0.2	0.0	0.1	0.2	0.2
A13	0.1	0.3	0.1	0.2	0.2	0.0	0.2	0.1	0.0	0.1	0.2	0.0	0.0	0.3	0.1	0.1	0.1	0.1	0.1	0.1	0.1
A14	0.0	0.1	0.2	0.0	0.2	0.1	0.1	0.0	0.1	0.0	0.1	0.1	0.1	0.1	0.1	0.2	0.0	0.0	0.2	0.0	0.0

*Step 6.* Next, we normalize the PF evaluation matrix Γ using the expression (49) and formulate the PF decision matrix (see Table 10).

**Table 10:** PF decision matrix

Mobile wallets	C1			C2			C3			C4			C5			C6			C7		
	( +)			( +)			( +)			( +)			( +)			( +)			( −)		
	μ	η	ν	μ	η	ν	μ	η	ν	μ	η	ν	μ	η	ν	μ	η	ν	μ	η	ν
A1	0.5	0.3	0.1	0.7	0.1	0.1	0.7	0.2	0.0	0.3	0.5	0.0	0.6	0.2	0.0	0.4	0.3	0.2	0.5	0.2	0.2
A2	0.4	0.4	0.0	0.7	0.1	0.0	0.7	0.0	0.1	0.8	0.0	0.0	0.4	0.2	0.2	0.7	0.1	0.0	0.5	0.1	0.2
A3	0.9	0.1	0.0	0.9	0.1	0.0	0.9	0.1	0.0	0.9	0.1	0.0	0.8	0.2	0.0	0.7	0.3	0.0	0.6	0.1	0.3
A4	0.3	0.3	0.2	0.6	0.1	0.0	0.7	0.2	0.0	0.6	0.3	0.0	0.7	0.1	0.0	0.4	0.4	0.0	0.3	0.3	0.2
A5	0.0	0.1	0.1	0.0	0.3	0.0	0.0	0.5	0.0	0.0	0.3	0.2	0.0	0.3	0.1	0.0	0.2	0.1	0.0	0.2	0.2
A6	0.2	0.0	0.3	0.1	0.1	0.3	0.1	0.2	0.3	0.1	0.2	0.3	0.0	0.1	0.4	0.3	0.1	0.1	0.0	0.0	0.4
A7	0.0	0.1	0.1	0.1	0.0	0.1	0.0	0.1	0.1	0.1	0.0	0.1	0.1	0.0	0.1	0.1	0.0	0.0	0.0	0.1	0.1
A8	0.0	0.4	0.1	0.1	0.3	0.1	0.2	0.2	0.1	0.0	0.4	0.1	0.1	0.3	0.1	0.0	0.3	0.1	0.1	0.3	0.0
A9	0.0	0.3	0.2	0.2	0.2	0.1	0.1	0.1	0.0	0.2	0.1	0.0	0.2	0.0	0.0	0.4	0.0	0.0	0.1	0.0	0.1
A10	0.0	0.4	0.3	0.3	0.0	0.2	0.0	0.3	0.0	0.0	0.4	0.0	0.0	0.3	0.1	0.0	0.3	0.0	0.0	0.2	0.2
A11	0.4	0.0	0.3	0.4	0.1	0.1	0.5	0.0	0.1	0.1	0.2	0.1	0.1	0.0	0.3	0.5	0.0	0.0	0.3	0.0	0.3
A12	0.2	0.4	0.1	0.3	0.3	0.0	0.3	0.2	0.0	0.2	0.2	0.0	0.1	0.1	0.3	0.2	0.2	0.0	0.2	0.2	0.1
A13	0.1	0.3	0.1	0.2	0.2	0.0	0.2	0.1	0.0	0.1	0.2	0.0	0.0	0.3	0.1	0.1	0.1	0.1	0.1	0.1	0.1
A14	0.0	0.1	0.2	0.0	0.2	0.1	0.1	0.0	0.1	0.0	0.1	0.1	0.1	0.1	0.1	0.2	0.0	0.0	0.0	0.0	0.2

*Step 7*. The average solution is calculated using the expression (50) with respect to each criterion.

For example, $$\overline{\gamma }_{1} = \mu_{{{{\rm i}}1}}^{\prime } ,\upeta_{{{{\rm i}}1}}^{\prime } ,\upupsilon_{{{{\rm i}}1}}^{\prime }$$

$$\mu_{i1}^{{\prime }} = \frac{1}{14}\mathop \sum \limits_{i = 1}^{14} \mu_{i1} = \, 0.2143;\quad \upeta_{i1}^{{\prime }} = \frac{1}{14}\mathop \sum \limits_{i = 1}^{14} \upeta_{i1} = 0.229;\quad \upupsilon_{i1}^{{\prime }} = \frac{1}{14}\mathop \sum \limits_{i = 1}^{14} \upupsilon_{i1} = 0.15$$

Table 11 provides the values of the average solutions.

**Table 11:** Average solutions

Avg. sol	C1			C2			C3			C4			C5			C6			C7		
	0.21	0.23	0.15	0.33	0.15	0.08	0.32	0.16	0.06	0.24	0.21	0.06	0.23	0.16	0.13	0.29	0.16	0.04	0.19	0.13	0.19

*Step 8*. Next, we calculate the PDA and NDA values using Eqs. (51–52)

For example,

$${\text{D}}_{11}^{ + } = \frac{{{{\rm Max}} \left( {0, \left( {{{\rm S}}\left( {\gamma_{11} } \right) - {\text{S}}\left( {\overline{\gamma }_{1} } \right)} \right)} \right)}}{{{{\rm S}}\left( {\overline{\gamma }_{1} } \right)}} = 5.22;\quad {\text{D}}_{51}^{ - } = \frac{{{{\rm Max}} \left( {0, \left( {{{\rm S}}\left( {\overline{\gamma }_{1} } \right) - {\text{S}}\left( {\gamma_{51} } \right)} \right)} \right)}}{{{{\rm S}}\left( {\overline{\gamma }_{1} } \right)}} = 2.56$$

*Step 9*. Calculation of the grey correlation coefficient (GC) values using Eqs. (53–54) wherein we consider ξ = 0.5.

For example,

$${\text{GP}}_{11} = \frac{0.00 + 0.5 \times 13.00}{{\left| {13.00 - 5.22} \right| + 0.5 \times 13.00}} = 0.455;\quad {\text{GN}}_{11} = \frac{0.00 + 0.5 \times 5.67}{{\left| {5.67 - 0.00} \right| + 0.5 \times 5.67}} = 0.333$$

*Step 10.* Calculation of the average weighted GC values by applying expressions (55–56)

*Steps 11–12*. Normalization of the average weighted GC values and final ranking using Eqs. (57–59). Table 12 summarizes the calculated results for steps 10–12 and final ranking results.

**Table 12:** Grey-correlation based ranking of wallets

Mobile wallets	GCP	GCN	N-GCP	N-GCN	GAS	Rank
A1	0.0750	0.0483	0.5252	0.5897	0.5575	4
A2	0.0950	0.0476	0.6650	0.5954	0.6302	2
A3	0.1429	0.0476	1.0000	0.5954	0.7977	1
A4	0.0749	0.0476	0.5241	0.5954	0.5598	3
A5	0.0476	0.0945	0.3333	0.1973	0.2653	13
A6	0.0476	0.1177	0.3333	0.0000	0.1667	14
A7	0.0476	0.0708	0.3333	0.3987	0.3660	9
A8	0.0487	0.0811	0.3407	0.3111	0.3259	11
A9	0.0507	0.0586	0.3547	0.5022	0.4284	7
A10	0.0476	0.0825	0.3333	0.2992	0.3163	12
A11	0.0538	0.0556	0.3764	0.5273	0.4518	5
A12	0.0497	0.0527	0.3482	0.5526	0.4504	6
A13	0.0476	0.0602	0.3333	0.4885	0.4109	8
A14	0.0476	0.0786	0.3333	0.3323	0.3328	10

It is seen that PhonePe (A3), Google Pay (A2), Amazon Pay (A4) and PayTM (A1) hold top positions. From the responses, it is revealed that user friendliness, wide variety of use and familiarity and awareness about the products help reducing the uncertainty factors and obtaining positive impressions from the users. On the other hand, JioMoney (A8), Mobikwik (A10), Freecharge (A5) and BHIM Axis Pay (A6) are the botton-level performers for not so attractive on previously mentioned factors.

## Validation and sensitivity analysis

The results obtained by using a particular MCDA technique require to be rational, reliable, bias free, and stable (Mukhametzyanov 2021; Baydaş and Elma 2021). The ranking by a MCDA method undergoes variations in preferential ordering because of several reasons such as change in the criteria weights, selection of normalization schemes, inclusion of a new and/or exclusion of an existing alternative(s), presence of a considerable amount of subjectivity, selection of appropriate criteria and defining their true nature (Bobar et al. 2020; Pamučar et al. 2016, 2019; Ecer and Pamucar 2020; Biswas et al. 2019; Gupta et al. 2019; Biswas and Pamucar 2020). As a result, in many cases, extant literature pointed out various drawbacks of MCDA techniques such as inconsistency in ranking (given a problem) between any two algorithms and/or among different experimental setups of a particular algorithm, and rank reversal phenomena (Žižović et al. 2020; Pamučar et al. 2017). Belton and Gear (Belton and Gear 1985) remarked that rank reversal is one of worst problem that lead highly inconsistent, illogical and wrong decisions using MCDA techniques. Therefore, it is imperative to check the validity and stability of the results obtained by using our proposed methodology. In this paper, we check the validity of results by comparing with the outcome of other algorithms. We then check the efficacy of our method with respect to the rank reversal problem. Finally, for examining the stability of the results we perform the sensitivity analysis. In the following sub-sections, we present the findings of all these tests.

### Comparison with results obtained from other MCDA frameworks

For comparing the results obtained from our method with that of other algorithms, we conduct two tests.

First, we perform the comparative ranking of the mobile wallets using the PF TOPSIS methodology used in Yang and He (2019).

Next, we use the actual score calculations of PFNs (Si et al. 2019) in the basic framework of EDAS method (Ghorabaee et al. 2015) for preferential ordering of the wallets. This provides an extension of the classical EDAS method in PF domain (ASPF-EDAS) which we use in our paper.

Table 13 shows that the ranking derived by using our GCPF-EDAS method is consistent with the results provided by PF-TOPSIS and ASPF-EDAS. Table 14 highlights that the rank correlations are strong and statistically significant.

**Table 13:** Comparison of ranking

Mobile wallets	Ranking results		
	GCPF-EDAS	PF-TOPSIS	ASPF-EDAS
A1	4	4	4
A2	2	2	2
A3	1	1	1
A4	3	3	3
A5	13	11	13
A6	14	14	14
A7	9	12	9
A8	11	8	10
A9	7	7	7
A10	12	10	12
A11	5	5	5
A12	6	6	6
A13	8	9	8
A14	10	13	11

**Table 14:** Rank correlation test I

Coefficient	Method	PF_TOPSIS	ASPF_EDAS
Kendall's τ	GCPF_EDAS	0.846**	0.978**
Spearman's ρ		0.921**	0.996**

^**^Correlation is significant at the 0.01 level (2-tailed)

### Rank reversal test

Rank reversal is typical issue vis-à-vis MCDA methodologies wherein the original ranking order of the alternatives gets changed with effect of inclusion of a new alternative or exclusion of an existing alternative (Pamučar et al. 2017; Belton and Gear 1985; Biswas et al. 2021a). In our paper, we perform the rank reversal test for the proposed GCPF-EDAS method by deleting a particular alternative, say A9 from the system given ξ = 0.5. We find the following result.

Original order:

$${\text{A}}3 \succ {\text{A}}2 \succ {\text{A}}4 \succ {\text{A}}1 \succ {\text{A}}11 \succ {\text{A}}12 \succ {\text{A}}9 \succ {\text{A}}13 \succ {\text{A}}7 \succ {\text{A}}14 \succ {\text{A}}8 \succ {\text{A}}10 \succ {\text{A}}5 \succ {\text{A}}6$$

Revised order (after deleting A9)

$${\text{A}}3 \succ {\text{A}}2 \succ {\text{A}}4 \succ {\text{A}}1 \succ {\text{A}}11 \succ {\text{A}}12 \succ {\text{A}}13 \succ {\text{A}}7 \succ {\text{A}}14 \succ {\text{A}}8 \succ {\text{A}}10 \succ {\text{A}}5 \succ {\text{A}}6$$

Therefore, we find that GCPF-EDAS does not suffer from the rank reversal problem.

### Sensitivity analysis

The sensitivity analysis is conducted to the effect of variations in the given conditions on the ranking result provided by a particular MCDA algorithm. In other word, the purpose of carrying out the sensitivity analysis is to ascertain the stability of the outcome of the MCDA methods (Pamučar and Ćirović 2015). The change in the criteria weights is one of the major sources of variations in the given conditions that affect the results of MCDA methods. Hence, one of the popular ways to carry out the sensitivity analysis is exchange of criteria weights (Önüt et al. 2009; Biswas and Anand 2020; Biswas et al. 2021b; Pramanik et al. 2021). In our paper, we follow this scheme which is demonstrated in the Table 15.

**Table 15:** Experiments for the sensitivity analysis

Criteria	C1	C2	C3	C4	C5	C6	C7
Cases	( +)	( +)	( +)	( +)	( +)	( +)	( −)
Original	0.14679	0.18349	0.12844	0.16514	0.13761	0.15596	0.08257
Exp 1	0.14679	**0.08257**	0.12844	0.16514	0.13761	0.15596	**0.18349**
Exp 2	0.14679	**0.12844**	**0.18349**	0.16514	0.13761	0.15596	0.08257
Exp 3	**0.16514**	0.18349	0.12844	**0.14679**	0.13761	0.15596	0.08257
Exp 4	0.14679	0.18349	**0.08257**	0.16514	0.13761	0.15596	**0.12844**

The bold cells indicate the exchange of criteria weights among them

**Fig. 1:**
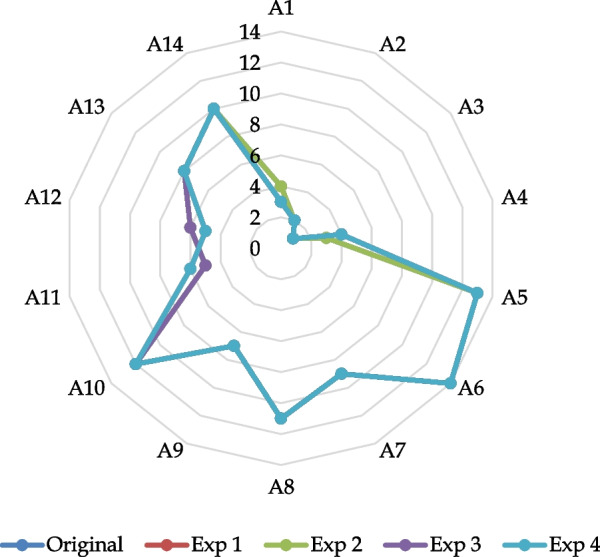
Results of the sensitivity analysis

We carry out the comparative ranking of the alternatives for each experimental cases such as Exp 1, Exp 2, Exp 3, and Exp 4. Table 16 summarizes the results and Table 17 shows the results of rank correlation test. We observe that GCPF-EDAS yields a stable ranking result as the ranking orders under different cases are significantly correlated with each other and with that of the original case. Figure 1 pictorially supports this finding and concludes that GCPF-EDAS performs considerably well in the sensitivity analysis.

**Table 16:** Comparison of ranking orders (sensitivity analysis)

Mobile wallets	Rank				
	Original	Exp 1	Exp 2	Exp 3	Exp 4
A1	4	3	4	3	3
A2	2	2	2	2	2
A3	1	1	1	1	1
A4	3	4	3	4	4
A5	13	13	13	13	13
A6	14	14	14	14	14
A7	9	9	9	9	9
A8	11	11	11	11	11
A9	7	7	7	7	7
A10	12	12	12	12	12
A11	5	6	5	5	6
A12	6	5	6	6	5
A13	8	8	8	8	8
A14	10	10	10	10	10

**Table 17:** Rank correlation test II

Coefficient	Case	Original	Exp1	Exp2	Exp3	Exp4
Kendall's τ	Original	1				
	Exp1	.956**	1			
	Exp2	1.000**	.956**	1		
	Exp3	.978**	.978**	.978**	1	
	Exp4	.956**	1.000**	.956**	.978**	1
Spearman's ρ	Original	1				
	Exp1	.991**	1			
	Exp2	1.000**	.991**	1		
	Exp3	.996**	.996**	.996**	1	
	Exp4	.991**	1.000**	.991**	.996**	1

**Correlation is significant at the 0.01 level (2-tailed)

Further, we move on to vary the values of ξ and examine the impact on the final ranking. Table 18 shows the comparative ranking (following usual procedural steps of our proposed framework) of M-wallets under study with varying values of ξ. We observe that GCPF-EDAS provides considerably similar ranking pattern even with varying ξ.

**Table 18:** Comparison of ranking orders (sensitivity analysis with varying ξ)

Mobile wallets	Ranking_ GCPF-EDAS		
	ξ = 0.5	ξ = 0.9	ξ = 0.1
A1	4	4	3
A2	2	2	2
A3	1	1	1
A4	3	3	4
A5	13	13	13
A6	14	14	14
A7	9	9	9
A8	11	10	12
A9	7	7	7
A10	12	12	11
A11	5	5	5
A12	6	6	6
A13	8	8	8
A14	10	11	10

## Conclusion and future scope

In this paper, we address a real-life problem of mobile wallet selection in the Indian context. We use the views of the users in this regard. We follow the fundamental framework of TAM vis-à-vis service quality dimensions for selection of criteria. We select a list of 14 popular mobile wallet service providers in India. These mobile wallets are used in various applications. Since, any subjective opinion based group decision-making involves a significant amount of uncertainty and impreciseness; most often deterministic models do not give appropriate results. Therefore, we carry out our analysis under uncertain environment using PFNs. Further due to an increasing level of uncertainty, past studies used the grey concept. In our work, we propose a GCPF-EDAS framework for comparative analysis. However, we also extend the fundamental algorithm of EDAS method in PF domain by using actual score based analysis. We investigate the stability and robustness of our method by comparing with the results of PF-TOPSIS and actual score based PF-EDAS method and carrying out a sensitivity analysis. We find that user friendliness, features, and awareness are some of the factors that influence the final ranking.

However, this paper has some scope for future work too. Firstly, this paper presents a small-scale nonparametric analysis. The same may be further tested by carrying out a large scale empirical analysis. Secondly, the interrelationship among the criteria may be tested by using causal models. Thirdly, the influence of the individual criterion on the pre-purchase, purchase and post-purchase decisions shall be examined. Fourthly, in the present study, we have selected the criteria in line with the findings of the prior research following the fundamental framework of TAM and UTAUT. However, there is a possibility to utilize the theoretical lens of TAM and UTAUT and other theories of consumer behaviour such as Consumer Experience (CX), Brand Experience (BX) for exploring the criteria for comparing the M-Wallets and then carry out the comparative analysis. Fifthly, one of the major future scopes of the present paper is to consider objective measurements for the comparative evaluation of the M-Wallets. In this study, we have considered objective criteria such as no of end users, subscription or transactional value per year, market share, growth in the customer bases, variety of services in terms of product offerings, transactional cost value etc. One future study may use these objective attributes to compare the M-Wallets and may carry out a comparative analysis of the rankings based on objective and subjective information respectively. Sixthly, we are also curious to see the behavioural pattern of the digitally low-literate consumers vis-à-vis selection of the mobile wallets as the government of India intends to expand the usage of digital payments in the long run for financial inclusion and prevention of corruption towards a vision of creating a cashless economy. Seventhly, technically the GCPF-EDAS framework can be used in solving various other problems involving multiple criteria. The procedural steps of this method may be followed to extend several other MCDA methods with imprecise information. Eight, in this paper we have used Type I PFS along with GC. However, a future attempt may include Type II PFS wherein membership function is itself fuzzy in nature. In that case, a further granular analysis at individual DM level may be carried out. Finally, apart from PFS, our model may be extended using the Neutrosophic Fuzzy Sets (NFS) which is a generalization of PFS. A possible future study may look at the possibility to extend EDAS method with a combination of NFS and GC.

Nevertheless, we believe that the above-mentioned future scopes do not undermine the usefulness of this study as within our best possible search we could notice that a work of this kind is quite rare. Further, the outcome of this paper shall provide necessary impetus to the corporate decision-makers and the organizations for formulating their future courses of actions. Our framework provides wide options (positive, negative, neutral and refusal membership choices and flexibility in selecting differentiating coefficient values) according to their preferences to decision-makers. Therefore, the proposed method may be applied to solve various real-life global issues such as portfolio selection for stock market investments, facility location selection for global operations, comparison of promotional strategies for product launching in global markets among others. For the problem of M-Wallet selection, our framework may be applied to compare globally accepted solutions taking opinions of the consumers from various countries. However, the choice criteria may vary in some countries (for example, developed nations and developing countries like India). This is perhaps a global limitation of the model used for comparing M-Wallets in India which may be solved by adding a prior Delphi study in conjunction with our GCPF-EDAS algorithm.

## Abbreviations

TAM: Technology Acceptance Model
TRA: Theory of reasoned action
TPB: Theory of planned behaviour
UTAUT: Unified theory of acceptance and use of technology
M-Wallet: Mobile wallet
MCDA: Multi-criteria decision analysis
EDAS: Evaluation based on distance from average solution
GCPF-EDAS: Grey correlation-based Picture Fuzzy-Evaluation based on Distance from Average Solution
TOPSIS: The Technique for Order of Preference by Similarity to Ideal Solution
VIKOR: VlseKriterijumska Optimizacija I Kompromisno Resenje
PDA: Positive distance from the average
NDA: Negative distance from the average
PF: Picture fuzzy
PFS: Picture fuzzy sets
IFS: Intuitionistic fuzzy sets
PFN: Picture fuzzy numbers
GC: Grey correlation
GRA: Grey relational analysis
GOI: Government of India
POS: Point of sale
DM: Decision makers
DMU: Decision making units

## Footnotes

1 https://main.mohfw.gov.in/digital-payment(last accessed September 28, 2022).

2 https://www.rbi.org.in/Scripts/PublicationVisionDocuments.aspx?Id=1202 (last accessed September 28, 2022).
